# Supplementary material for: Understanding the neural code of stress to control anhedonia
Source: Nature. 2024 Dec 4;637(8046):654–62. doi: 10.1038/s41586-024-08241-y (PMC11735319; doi:10.1038/s41586-024-08241-y)
Supplement: Supplementary file 1 — Reporting Summary [file 41586_2024_8241_MOESM1_ESM.pdf]

nature portfolio

Corresponding author(s): Kheirbek

Last updated by author(s): Sep 25, 2024

Reporting Summary

Nature Portfolio wishes to improve the reproducibility of the work that we publish. This form provides structure for consistency and transparency in reporting. For further information on Nature Portfolio policies, see our Editorial Policies and the Editorial Policy Checklist.

Statistics

For all statistical analyses, confirm that the following items are present in the figure legend, table legend, main text, or Methods section.

n/a	Confirmed	
☐	☒	The exact sample size (*n*) for each experimental group/condition, given as a discrete number and unit of measurement
☐	☒	A statement on whether measurements were taken from distinct samples or whether the same sample was measured repeatedly
☐	☒	The statistical test(s) used AND whether they are one- or two-sided<br>*Only common tests should be described solely by name; describe more complex techniques in the Methods section.*
☒	☐	A description of all covariates tested
☐	☒	A description of any assumptions or corrections, such as tests of normality and adjustment for multiple comparisons
☐	☒	A full description of the statistical parameters including central tendency (e.g. means) or other basic estimates (e.g. regression coefficient) AND variation (e.g. standard deviation) or associated estimates of uncertainty (e.g. confidence intervals)
☐	☒	For null hypothesis testing, the test statistic (e.g. *F*, *t*, *r*) with confidence intervals, effect sizes, degrees of freedom and *P* value noted<br>*Give P values as exact values whenever suitable.*
☒	☐	For Bayesian analysis, information on the choice of priors and Markov chain Monte Carlo settings
☒	☐	For hierarchical and complex designs, identification of the appropriate level for tests and full reporting of outcomes
☐	☒	Estimates of effect sizes (e.g. Cohen's *d*, Pearson's *r*), indicating how they were calculated

Our web collection on statistics for biologists contains articles on many of the points above.

Software and code

Policy information about availability of computer code

Data collection	Neuropixels data were collected using SpikeGLX (v20220101-phase30). Behavioral data were collected using Arduino and CoolTerm 1.9. Facial and limb movement videos were recorded using the MATLAB Image Acquisition Toolbox.
Data analysis	Data were analyzed using custom codes in MATLAB and Python, including the use of the following: scikit-learn, Kilosort 2, Kilosort 4, Phy2, DeepLabCut, SSM toolbox (https://github.com/lindermanlab/ssm), and Allen CCF (https://github.com/cortex-lab/allenCCF). Code available on https://github.com/mkheirbek.

For manuscripts utilizing custom algorithms or software that are central to the research but not yet described in published literature, software must be made available to editors and reviewers. We strongly encourage code deposition in a community repository (e.g. GitHub). See the Nature Portfolio guidelines for submitting code & software for further information.

Data

Policy information about availability of data

All manuscripts must include a data availability statement. This statement should provide the following information, where applicable:

- Accession codes, unique identifiers, or web links for publicly available datasets
- A description of any restrictions on data availability
- For clinical datasets or third party data, please ensure that the statement adheres to our policy

All source data is provided with this paper. The raw electrophysiology data will be provided upon reasonable request.

Research involving human participants, their data, or biological material

Policy information about studies with human participants or human data. See also policy information about sex, gender (identity/presentation), and sexual orientation and race, ethnicity and racism.

Reporting on sex and gender	N/A
Reporting on race, ethnicity, or other socially relevant groupings	N/A
Population characteristics	N/A
Recruitment	N/A
Ethics oversight	N/A

Note that full information on the approval of the study protocol must also be provided in the manuscript.

Field-specific reporting

Please select the one below that is the best fit for your research. If you are not sure, read the appropriate sections before making your selection.

☒ Life sciences ☐ Behavioural & social sciences ☐ Ecological, evolutionary & environmental sciences

For a reference copy of the document with all sections, see nature.com/documents/nr-reporting-summary-flat.pdf

Life sciences study design

All studies must disclose on these points even when the disclosure is negative.

Sample size	Sample sizes for animals were based on previous studies using the techniques described (e.g. Krishnan et al., Cell, 2007). No further sample size calculations were used.
Data exclusions	For DREADD experiments, animals with off-target expressions of viruses were excluded, in order to specifically analyze the effects of manipulating only the region of interest.
Replication	All recording and behavioral data were acquired from a minimum of 2 independently performed experimental cohorts. Data were randomly subdivided during analysis to ensure reproducibility across subsets.
Randomization	Mice were randomly assigned to control vs. CSDS groups before CSDS training. For DREADD experiments, mice were randomly assigned to mCherry vs. hM3Di groups at time of surgery.
Blinding	Experimenters were blind to the condition and group assignments of mice. The same analysis pipeline was applied to all animals.

Reporting for specific materials, systems and methods

We require information from authors about some types of materials, experimental systems and methods used in many studies. Here, indicate whether each material, system or method listed is relevant to your study. If you are not sure if a list item applies to your research, read the appropriate section before selecting a response.

Materials & experimental systems

n/a	Involved in the study	
☒	☐	Antibodies
☒	☐	Eukaryotic cell lines
☒	☐	Palaeontology and archaeology
☐	☒	Animals and other organisms
☒	☐	Clinical data
☒	☐	Dual use research of concern
☒	☐	Plants

Methods

n/a	Involved in the study	
☒	☐	ChIP-seq
☒	☐	Flow cytometry
☒	☐	MRI-based neuroimaging

Animals and other research organisms

Policy information about studies involving animals; ARRIVE guidelines recommended for reporting animal research, and Sex and Gender in Research

Laboratory animals	Adult (8-12 weeks old) male and female C57BL/6J mice, and adult (5-6 months old) CD1 retired male breeder mice were used.
Wild animals	No wild animals were used in the study.
Reporting on sex	Both male and female mice were used.
Field-collected samples	No field-collected samples were used in the study.
Ethics oversight	All procedures were conducted in accordance with the NIH Guide for the Care and Use of Laboratory Animals and institutional guidelines.

Note that full information on the approval of the study protocol must also be provided in the manuscript.

Plants

Seed stocks	N/A
Novel plant genotypes	N/A
Authentication	N/A
